# iRoot SP Promotes Osteo/Odontogenesis of Bone Marrow Mesenchymal Stem Cells via Activation of NF-*κ*B and MAPK Signaling Pathways

**DOI:** 10.1155/2020/6673467

**Published:** 2020-12-24

**Authors:** Xiao Wu, Ming Yan, Jiamin Lu, Xingyun Ge, Yuzhi Li, Minxia Bian, Lin Fu, Jinhua Yu

**Affiliations:** ^1^Key Laboratory of Oral Diseases of Jiangsu Province, Institute of Stomatology, Nanjing Medical University, 136 Hanzhong Road, Nanjing 210029, China; ^2^Endodontic Department, School of Stomatology, Nanjing Medical University, 136 Hanzhong Road, Nanjing 210029, China

## Abstract

The regeneration of bone and tooth tissues, and related cellular therapies, has attracted widespread attention. Bone marrow mesenchymal stem cells (BMSCs) are potential candidates for such regeneration. iRoot SP is a premixed bioceramic root canal sealer widely used in clinical settings. However, the effect of iRoot SP on the biological features of BMSCs has not been elucidated. In the present study, we found that 0.2 mg/ml iRoot SP conditioned medium promoted osteo/odontogenic differentiation and enhanced mineralization of BMSCs without affecting the proliferative ability. Mechanistically, the NF-*κ*B and MAPK signaling pathways were activated in SP-treated BMSCs, and differentiation was inhibited when cultured with the specific inhibitor. Taken together, these findings demonstrate that iRoot SP promotes osteo/odontogenic differentiation of BMSCs via the NF-*κ*B and MAPK signaling pathways, which could provide a new theoretical basis for clinical applications of iRoot SP and a new therapeutic target for the regeneration of bone and tooth tissue in the future.

## 1. Introduction

The pulp tissue of teeth can be affected by trauma, dental caries, and anatomic variation, leading to clinical cases of pulpitis and periapical periodontitis [1]. Root canal treatment (RCT) is effective for removing infected dentin and pulp, promoting recovery from inflammation and preventing reinfection of the tooth's root and crown [2]. The purpose of a root canal filling is to form a complete 3D filling. Various materials, including zinc oxide eugenol, resin, glass ionomer, and calcium hydroxide, have been utilized as root canal sealers [3]. In recent years, bioceramic-based sealers have attracted attention [4].

iRoot SP (Innovative BioCeramix Inc, Vancouver, Canada), a ready-to-use injectable bioceramic, is an aluminum-free material composed mainly of calcium silicate. iRoot SP is premixed with inorganic ingredients (calcium silicate, calcium dihydrogen phosphate, calcium hydroxide, and filler), developer (zirconia), and nonaqueous thickener [5]. iRoot SP is suitable for matched-taper single cone obturation and lateral pressure filling technology. Further, it has been shown to have superior antibacterial activity, biocompatibility, cytocompatibility, high resistance to dislocation, and to promote healing of periapical tissues [6]. Recent research has examined the effects of iRoot SP on stem cells, such as human tooth germ stem cells [7] and human periodontal ligament cells [8].

When a sealer like iRoot SP is used, it is placed in the canal and confined there. However, it may be extruded through the apical foramen, or it may release chemicals into the periapical tissue [9]. Apical surgery is commonly performed for refractory chronic apical periodontitis. During this procedure, it is inevitable that sealers will come into contact with the surrounding tissues and cells. Due to their superior proliferative and multidirectional differentiation abilities, bone marrow mesenchymal stem cells (BMSCs) play a major role in stem cell therapy. Due to their biological properties, partially shared gene expression, and similar factor regulation [10], BMSCs can serve as alternative sources for dental stem cells [11]. In addition, BMSCs reportedly have the ability to migrate to the root canal and be involved in pulp formation. Thus, BMSCs may have applications for pulp regeneration [12]. Various factors, including adiponectin [13], bioactive materials [14], and microRNA [15], have been demonstrated to affect the osteogenic differentiation of BMSCs. However, the impact of iRoot SP on the biological activities of BMSCs remains unclear.

Diverse regulatory mechanisms, including the NF-*κ*B [16] and mitogen-activated protein kinase (MAPK) cascades [17], are known to participate in the multidirectional differentiation of MSCs. In 1986, the A B cell-specific inducible transcription factor was discovered by the research group of David Baltimore and identified as nuclear factor-*κ*B (NF-*κ*B) [18]. The NF-*κ*B family is essential for coordinating multiple cellular responses, including cellular proliferation [19], differentiation [20], and apoptosis [21]. MAPKs, which form a serine-threonine kinases family, are indispensable for multitudinous physiologic processes [22]. Many interactions between the two signaling pathways have been reported.

In the present study, we examined the effect of iRoot SP on BMSCs and the molecular mechanisms of any identified effects. The results revealed that iRoot SP promotes the osteo/odontogenic capacity of BMSCs via the NF-*κ*B and MAPK signaling cascades.

## 2. Materials and Methods

### 2.1. Purification and Growth of BMSCs

Bone marrow stromal cells were isolated from two-week-old male Sprague–Dawley rats obtained from the Experimental Animal Centre, Nanjing Medical University, China, as previously described [23]. All protocols were carried out in accordance with the guidelines of the Animal Experiment Committee of Nanjing Medical University. Briefly, rat femurs and tibiae were aseptically excised. The marrow cell suspension was flushed out using alpha minimum essential medium (*α*-MEM, Gibco, Life Technologies, Grand Island) and then centrifuged. Cells were planted in 60 mm culture dishes and incubated in *α*-MEM with 10% fetal bovine serum (FBS; Gibco, Grand Island), 100 U/ml penicillin, and 100 mg/ml streptomycin at 37°C in 5% CO_2_. The medium was replaced every two days. Cells were digested and passaged at a ratio of 1 : 3 once they reached 80-85% confluence. Cells at 2–4 passages were utilized for the assays in this study.

### 2.2. Preparation of iRoot SP Conditioned Medium

iRoot SP (Innovative BioCeramix Inc., Vancouver, Canada) was solidified at 37°C in a 100% humidified atmosphere with 5% CO_2_. The biomaterials were dried for one day, ground into powder, and filtered through a 45 *μ*m strainer. Next, they were mixed with *α*-MEM at a 20 mg/ml concentration and vigorously vortexed. The gathered solution was incubated for three days at 37°C to generate its bioactive contents. The supernatant was then filtered and mixed with *α*-MEM to form the SP-conditioned medium.

### 2.3. Flow Cytometry (FCM)

BMSCs were grown in complete medium and MEM containing 2 mg/ml SP for three days. The cells were harvested, fixed using prechilled alcohol, and stored at 4°C for 24 h in a dark room. After rinsing with PBS, the cell cycle fractions (G0G1/S/G2M phases) were determined using a FACSCalibur flow cytometer (BD Biosciences, CA, USA). These procedures were performed in triplicate.

### 2.4. Cell Counting Kit 8 (CCK8) Assessment

Cell proliferation was determined using the Cell Counting Kit-8 (CCK-8, Dojindo, Tokyo, Japan). BMSCs were planted into 96-well plates (Corning, USA) at a titer of 3 × 10^3^ cells per well and incubated for 24 h. The cells were then grown in complete medium or SP. At days 0, 1, 3, 5, 7, and 9, the medium was replaced with 10 *μ*L CCK-8 reagent for 2 h. The optical density (OD) values were measured at 450 nm.

### 2.5. Alkaline Phosphatase (ALP) Enzyme Activity and Staining

Alkaline phosphatase (ALP) enzyme activity was determined using a commercially available kit (Beyotime, China) in line with the manufacturer's instructions and standardized to the total protein titer. Protein was examined using a BCA kit (Beyotime, Shanghai, China). Cells were fixed in 4% paraformaldehyde for 20 mins, rinsed thrice, and stained using the ALP Staining Kit (Beyotime, China) according to the manufacturer's protocol.

### 2.6. Alizarin Red Staining and Quantification

Alizarin Red staining was used to investigate mineralization. On day 14, BMSCs were fixed for 30 min using 75% ethyl alcohol and dyed with 2% Alizarin Red (Sigma-Aldrich). Specimens were then visualized using a microscope. Calcified nodules were quantified by 10% cetylpyridinium chloride (CPC), and its absorbance was measured at 560 nm. The calcium denseness was normalized to the total protein content.

### 2.7. Western Blot

To investigate osteo/odontogenic differentiation, BMSCs were harvested with or without SP-treatment at days 0, 3, and 7. Cells were then treated with SP at 0, 30, 60, and 90 min and 0, 15, 30, and 60 min to measure the participation of the NF-*κ*B and MAPK cascades, respectively. For the NF-*κ*B pathway, cytoplasmic and nuclear proteins were extracted using a Keygen Kit (Keygen Bio-Tech, Nanjing, China) at the relevant time. To determine the effects of the repressors on the NF-*κ*B and MAPK signaling cascades, we pretreated the cells with the inhibitors (namely, BMS345541 targeting NF-*κ*B, U0126 targeting ERK, SP600125 targeting JNK, and SB203580 targeting P38) for 2 h and then cultured the cells with SP for 30 min or 15 min, respectively. BMSCs were lysed in RIPA buffer (Beyotime, China) with 1 mM phenylmethylsulfonyl fluoride on ice. The protein was separated by 10% sodium dodecyl sulfate-polyacrylamide gel electrophoresis and transfer embedded onto PVDF membranes (Millipore). We utilized 5% BSA to block the membranes for 2 h at room temperature, followed by overnight incubation with primary antibodies (DSPP (BS70836; Bioworld, USA), OPN (ab8448, Abcam, UK), RUNX2 (ab76956, Abcam, UK), OSX (ab22552, Abcam, UK), P65 (#8242, Cell Signaling Technology, USA), p-P65 (#3033, Cell Signaling Technology, USA), I*κ*B*α* (#4814, Cell Signaling Technology, USA), p-I*κ*B*α* (#2859, Cell Signaling Technology, USA), JNK (#9252, Cell Signaling Technology, USA), p-JNK (#9255, Cell Signaling Technology, USA), p38 (#8690, Cell Signaling Technology, USA), p-p38 (#4511, Cell Signaling Technology, USA), ERK (#4695, Cell Signaling Technology, USA), p-ERK (#4370, Cell Signaling Technology, USA), Histone H3 (Bioworld), and GAPDH (#2118, Cell Signaling Technology, USA)). After washing with TBST, the membranes were incubated with secondary antibodies for 1 h and rinsed again with TBST for 30 min. Finally, the membranes were scanned using the Western Blot Imaging System (GE Healthcare, Beijing, China), and protein expression was quantified using the ImageJ software.

### 2.8. Real-Time RT-qPCR

TRIzol reagent (Invitrogen, NY, USA) was used to purify the total RNA (tRNA). Subsequently, tRNA was converted to cDNA via reverse transcription by employing the PrimeScript RT Master Mix kit (TaKaRa Biotechnology, China). Next, RT-PCR was performed using SYBR Green PCR master mix (Roche, Indianapolis, IN) on an ABI 7300 real-time PCR system. The thermal cycler was set at 95°C for 30 s, 40 cycles at 95°C for 5 s, and 60°C for 31 s. We utilized GAPDH to normalize the expression of the osteo/odontogenic genes (namely, *Osx*, *Alp*, *Runx2*, *Opn*, and *Dspp*), which was quantified using the 2^−*ΔΔ*CT^ approach. The sequences of the utilized primers are provided in Table 1.

### 2.9. Immunofluorescence Staining

BMSCs were seeded onto 10 mm 2 sterile glass coverslips and grown for 72 h. Next, they were fixed using 4% PFA for 30 min and permeabilized using Triton X-100 solution for 12 min. We employed normal goat serum (DCS/BioGenex, Hamburg, Germany) to block the cells for 30 min at 37°C, followed by overnight incubation at 4°C with the primary antibodies against ALP, RUNX2, P65, p-JNK, p-p38, and p-ERK. After incubation, cells were conjugated with secondary antibodies in the dark for 1 h. We counterstained the nuclei with DAPI (Beyotime, China), which were then inspected under an inverted fluorescence microscope.

### 2.10. Statistical Analyses

Each test was performed in triplicate. Data are reported as the mean ± SD. Results were inspected by one-way ANOVA or Student's *t*-test. The level of statistical significance was set as *p* ≤ .05. Statistical analyses were performed using the SPSS 20.0 software.

## 3. Results

### 3.1. Optimal Concentration of iRoot SP

**Figure 1:**
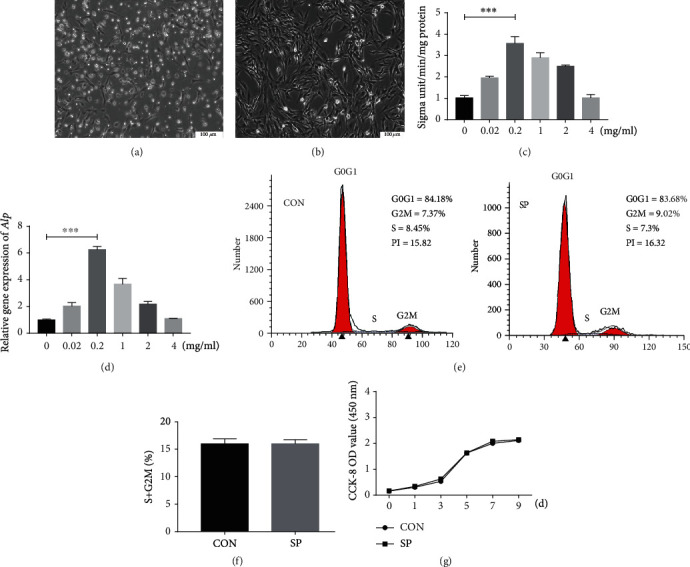
Optimum dose and effects of iRoot SP on BMSC proliferation. (a) Primary BMSCs with fibroblast- or spindle-like morphology. (b) Cells in passage 3. Scale bar = 100 *μ*m. (c) ALP activity of BMSCs cultured with different concentrations of SP at day 5. ^∗∗∗^*p* < .001. (d) iRoot SP at 0.2 mg/ml greatly promoted the ALP mRNA expression in BMSCs after 5 days compared with the control group. ^∗∗∗^*p* < .001. (e, f) Flow cytometry (FCM) analysis for control and SP-treated BMSCs. (g) After 9 days, the CCK-8 assay showed no remarkable differences in cellular proliferation between 0.2 mg/ml SP-treated BMSCs and untreated BMSCs.

Primary BMSCs cultivated for three days are shown in Figure 1(a). The cells had a spindle-shaped morphology (Figure 1(b)). BMSCs treated with 0.2 mg/ml SPshowed the highest upregulation of ALP enzyme activity in comparison with the other groups (*p* < .001; Figure 1(c)). The ALP gene expression revealed that BMSCs cultured with 0.2 mg/ml SP group had the highest expression (*p* < .001; Figure 1(d)). As a result, 0.2 mg/ml was chosen as the optimal concentration.

### 3.2. Effects of SP on BMSC Proliferation

Flow cytometry revealed a negligible difference in the proliferative index (PI = G2M + S) between the SP group and the control group (Figures 1(e) and 1(f)). The CCK-8 assay demonstrated that proliferation of BMSCs was not affected by culturing with SP (Figure 1(g)).

### 3.3. iRoot SP Enhanced Osteo/Odontogenic Differentiation of BMSCs

**Figure 2:**
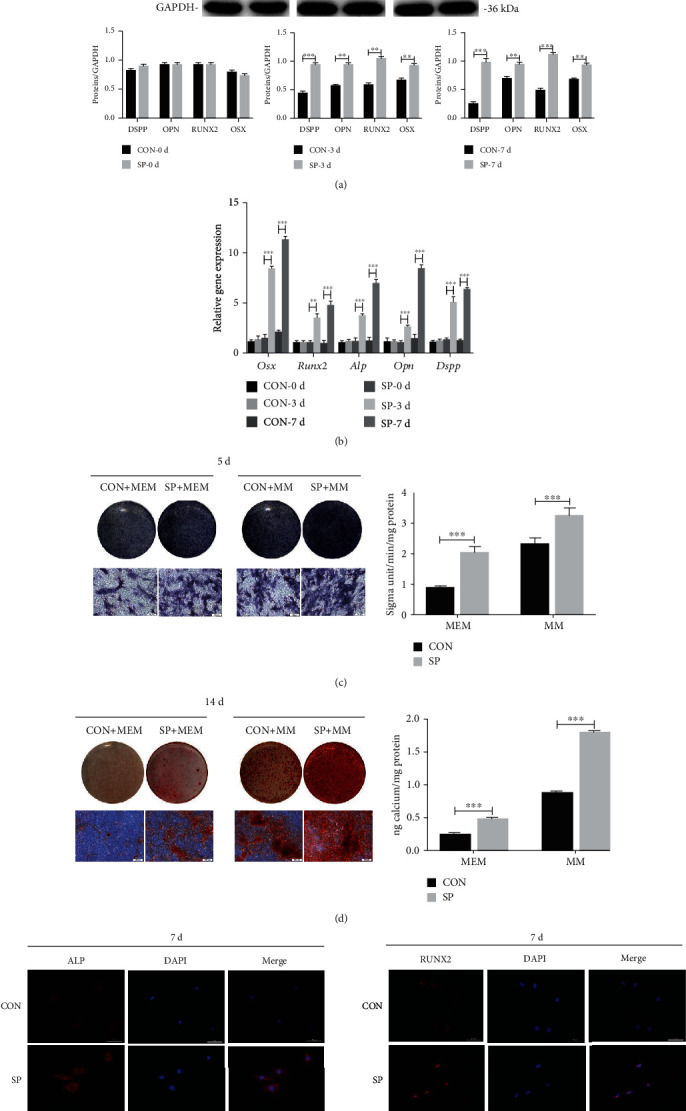
Impact of SP on osteo/odontogenic differentiation of BMSCs. (a) Western blot was used to investigate DSPP, OPN, RUNX2, and OSX protein expression at days 0, 3, and 7. Gray scale inspection of protein bands by ImageJ. ^∗∗^*p* < .01, and ^∗∗∗^*p* < .001. (b) RT-PCR demonstrated the relative gene expression in the control group, as well as the SP group, at days 0, 3, and 7. ^∗∗^*p* < .01, and ^∗∗∗^*p* < .001. (c) ALP staining and ALP activity for the control group, SP group, MM group, and MM + 0.2 mg/ml SP group at day 7. Scale bar = 100 *μ*m. ^∗∗∗^*p* < .001. (d) Figures of Alizarin Red staining and CPC assay in different groups. Scale bar = 100 *μ*m. ^∗∗∗^*p* < .001. (e) Immunofluorescence staining of ALP and RUNX2 in SP-treated BMSCs.

Western blot was used to assess the impact of SP on the osteo/odontogenic differentiation of BMSCs. The relative contents of dentin sialophosphoprotein (DSPP), osterix (OSX), osteopontin (OPN), and runt-related transcription factor 2 (RUNX2) were upregulated under SP treatment (Figure 2(a); *p* < .01). The gene expression levels of *Dspp*, *Opn*, *Runx2*, *Alp*, and *Osx* were also enhanced (Figure 2(b); *p* < .01). ALP staining and quantitation revealed that SP elevated the expression levels in BMSCs with or without the osteogenic differentiation medium (Figure 2(c); *p* < .001). Significantly, more mineralized nodules were observed after incubation with SP for 14 days (Figure 2(d); *p* < .001). Fluorescence imaging revealed a similar trend for the ALP and RUNX2 expression (Figure 2(e); *p* < .001). Together, these findings indicated that SP promoted the osteo/odontogenic differentiation of BMSCs.

### 3.4. iRoot SP Activated the NF-*κ*B Pathway in BMSCs

**Figure 3:**
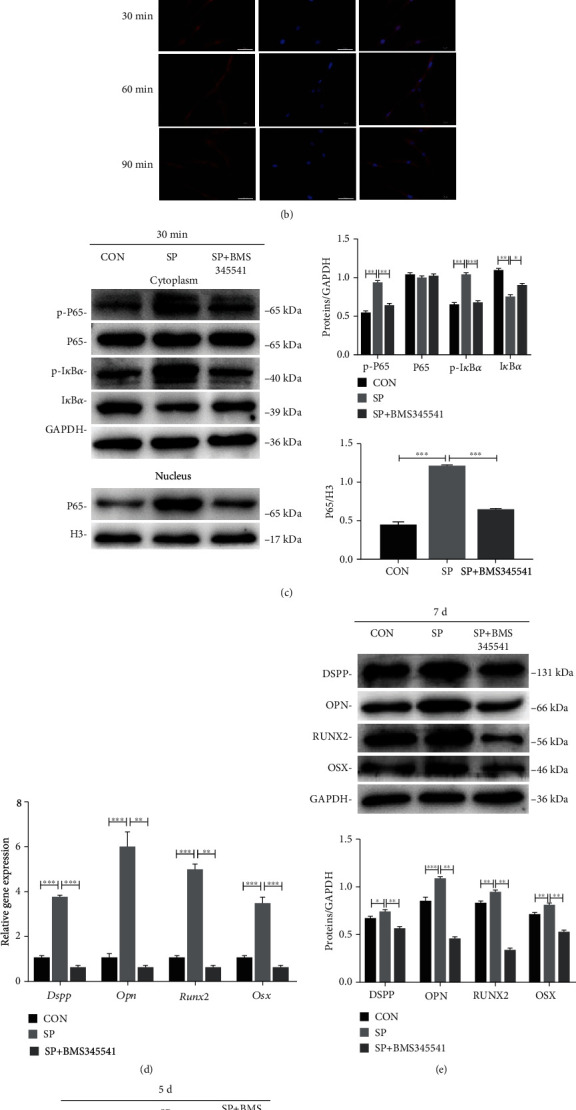
Impact of iRoot SP on the NF-*κ*B cascade of BMSCs. (a) Protein expression of I*κ*B*α*, p-I*κ*B*α*, P65, and p-P65 in the cytoplasm and nuclear P65 of SP-treated BMSCs at different time points. GAPDH was used as the internal control. Gray scale evaluations of protein by ImageJ. ^∗^*p* < .05, ^∗∗^*p* < .01, ^∗∗∗^*p* < .001. (b) Immunofluorescence assay of nuclear P65 at different time points. Scale bar = 50 *μ*m. (c) Protein titers of cytoplasm P65, p-P65, I*κ*B*α*, and p-I*κ*B*α*, as well as nuclear P65, in the NC group, SP group, and SP + BMS345541 group at 30 min. GAPDH and Histone 3 were used as internal controls. Gray scale assessment of protein bands by ImageJ. ^∗∗^*p* < .01, and ^∗∗∗^*p* < .001. (d) Gene expression of Dspp, Opn, Runx2, and Osx in the NC group, SP group, and SP + BMS345541 group at 30 min. ^∗∗^*p* < .01, ^∗∗∗^*p* < .001. (e) Protein expression of DSPP, OPN, RUNX2, and OSX in different groups at day 7. ^∗^*p* < .05, ^∗∗^*p* < .01, ^∗∗∗^*p* < .001. (f) ALP staining and ALP enzyme activity in the BMS345541 + SP group and SP group after 5 days. Scale bar = 100 *μ*m. ^∗∗^*p* < .01, and ^∗∗∗^*p* < .001. (g) Figures of Alizarin Red S staining of BMSCs in different groups. Absorbance was measured to evaluate the degree of mineralization. Scale bar = 100 *μ*m. ^∗∗^*p* < .01, and ^∗∗∗^*p* < .001.

To identify molecular changes in SP-treated BMSCs, cells were cultured with SP for 0, 30, 60, and 90 min, and the cytoplasm and nuclear proteins were then extracted. Analysis revealed that the phosphorylated levels of I*κ*B*α* and P65 were enhanced and reached peak levels at 30 min, whereas cytoplasmic I*κ*B*α* and nuclear P65 levels declined after 30 min of exposure but then increased (Figure 3(a); *p* < .05). Immunostaining revealed that nuclear P65 was translocated for 30 min (Figure 3(b)). Taken together, these results suggested that SP activated the NF-*κ*B pathway in BMSCs.

### 3.5. Inhibition of the NF-*κ*B Pathway Downregulated Osteo/Odontogenic Differentiation of SP-Treated BMSCs

To elucidate the function of the NF-*κ*B cascade in the differentiation of SP-treated BMSCs, a repressor (BMS345541) was utilized. The results revealed that phosphorylation of I*κ*B*α* and P65 was suppressed, whereas nuclear translocation of P65 was promoted. These results suggest that the NF-*κ*B pathway was blocked (Figure 3(c); *p* < .01). The gene expression of *Dspp*, *Opn*, *Runx2*, and *Osx* in the SP+ inhibitor arm was greatly suppressed relative to the SP arm (Figures 3(d) and 3(e), *p* < .01). Furthermore, the expression of ALP was low under treatment with BMS345541 (Figure 3(f); *p* < .001). A statistically significant decrease was also observed for Alizarin Red S staining in the SP + inhibitor arm (Figure 3(g); *p* < .001). Taken together, these findings demonstrated that the NF-*κ*B cascade was activated in SP-treated BMSCs.

### 3.6. iRoot SP Activated the MAPK Cascade in BMSCs

**Figure 4:**
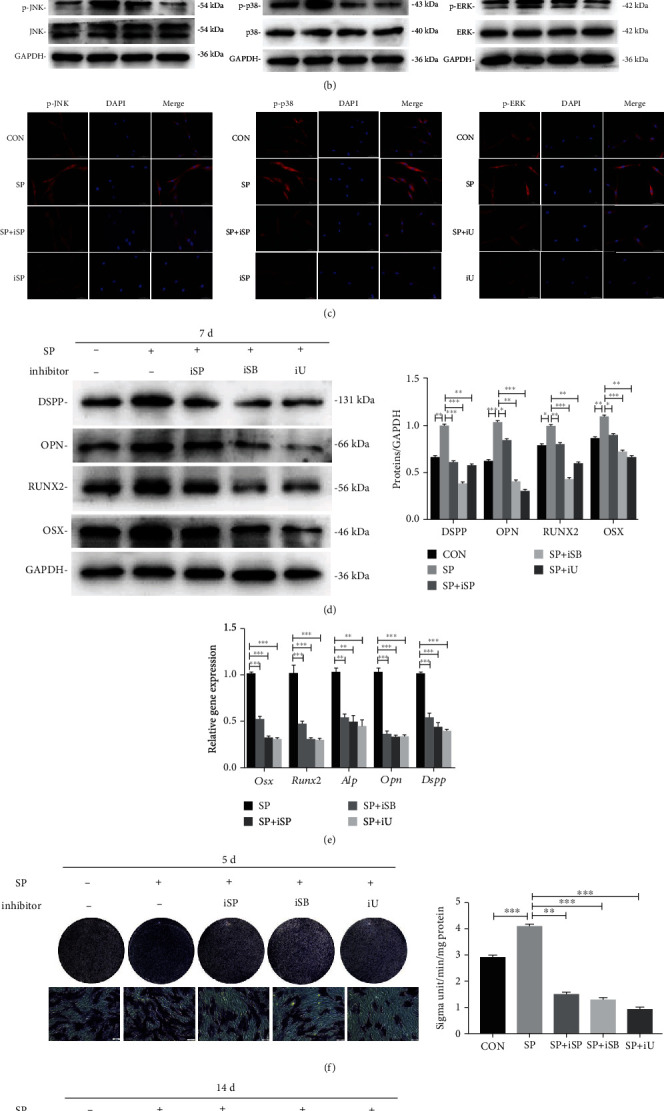
iRoot SP activated the MAPK cascades of BMSCs. (a) Protein titers of JNK, phospho-JNK, P38, p-p38, ERK, and p-ERK of SP-treated BMSCs and quantitative analysis of the ratios at different time points. ^∗^*p* < .05, and ^∗∗^*p* < .01. (b) The expression of MAPK-related proteins treated with cascade repressors (SP: SP600125, SB: SB203580, and U: U0126) was investigated by Western blot. ^∗∗^*p* < .01, and ^∗∗∗^*p* < .001. (c) Immunofluorescence staining of P-JNK, p-p38, and P-ERK after suppression of the MAPK cascades. Scale bar = 50 *μ*m. (d) Western blot assessment of osteo/odontogenic proteins in the different groups at day 7. Quantitative analysis of protein bands was performed using ImageJ. ^∗^*p* < .05, ^∗∗^*p* < .01, and ^∗∗∗^*p* < .001. (e) MRNA expression of osteo/odontogenic differentiation-associated genes was quantified by RT-PCR. ^∗^*p* < .05, ^∗∗^*p* < .01, and ^∗∗∗^*p* < .001. (f) ALP staining and quantification of ALP enzyme activity of BMSCs cultured with complete medium, SP, SP + SB203580, SP + SP600125, and SP + U0126 for 5 days. Scale bar = 100 *μ*m. ^∗∗^*p* < .01, and ^∗∗∗^*p* < .001. (g) Alizarin Red S staining and quantification analysis of calcium nodules in the different groups at different time points. Scale bar = 100 *μ*m. ^∗∗^*p* < .01, and ^∗∗∗^*p* < .001.

To inspect the contribution of the MAPK signaling cascade in the osteo/odontogenic differentiation of SP-treated BMSCs, proteins in the presence of SP at 0, 15, 30, and 60 min were verified by Western blot. The results showed that SP increased expression of p-JNK, p-p38, and p-ERK after 15 min but that expression subsequently decreased in a time-dependent manner (Figure 4(a), *p*< .01).

### 3.7. Inhibition of the MAPK Cascade Repressed the Osteo/Odontogenic Differentiation of SP-Treated BMSC

Western blot and immunofluorescence analysis indicated that, after treatment with the inhibitor (SP600125, SB203580, or U0126), expression of p-JNK, p-p38, and p-ERK expressions was inhibited. These results confirmed that the MAPK pathway was blocked (Figures 4(b) and 4(c); *p* < .01). The relative osteo/odontogenic markers were investigated in the SP+ inhibitor group and found to be notably decreased relative to the SP group (Figures 4(d) and 4(e); *p* < .01). As shown in Figures 4(f) and 4(g), the ALP level and calcium nodules exhibited lower expression values after cultivation with SP.

## 4. Discussion

BMSCs are a promising source of MSCs for tissue engineering regeneration methods, including regenerative therapies (RET) [24]. As BMSCs have the potential to differentiate into odontoblasts, they could regenerate dentin-like tissues [25]. Even if immature teeth without pulp and periapical tissue *in vitro*, bone-like is seen in the canal space after RET, which suggests that the cells may originate from bone marrow [26]. Previous studies have demonstrated the multidirectional differentiation, tissue differentiation potential [27, 28], and osteogenesis [29] of BMSCs.

iRoot SP is known to have good cytocompatibilities that improve mineralization of MG63 cells [30], enhance odontogenic differentiation of hTGSCs [7], promote osteoblastic differentiation of hPDLCs [8], and present no or low cytotoxicity to fibroblast cell lines [31]. Energy-Dispersive X-ray Spectroscopy (EDX) has been used to analyze the composition of iRoot SP, finding oxygen (O), calcium (Ca), zirconium (Zr), carbon (C), and silicon (Si). After the solubility test, EDX revealed a decline of Ca, O, and Si and upregulated Zr values [32]. Calcium plays a crucial role in regulating cellular biological behavior. Several studies have confirmed that the release of calcium improves proliferation and induces osteogenic differentiation of BMSCs [33]. In addition, calcium may activate the ERK and p38 MAPK pathways in osteoblasts and initiate the Ras–MAPK signaling pathway by recruiting Src kinases and Sh tyrosine phosphorylated [34]. Calcium can trigger various signaling pathways, such as the NF-*κ*B pathway, through calcium-sensing receptors (CaSR) [35]. The movement of silicon can promote biomineralization [36] and increase osteogenic activities [37]. In general, calcium and silicon ions released by calcium silicate–based materials influence cellular biological behavior [38]. These eluates simulate clinical conditions in which cells and materials make indirect contact through blood clots [39] and can be conveniently observed and easily analyzed [40]. In the current study, the material eluates were prepared following ISO 10993-538 [41] as previously described [42].

ALP, as an early biosignature for osteo/odontogenic differentiation, has a close relationship with mineralization [43]. A concentration of 0.2 mg/ml iRoot SP was found to be optimal, based on the peak activity and mRNA levels of ALP. However, the proliferative ability of BMSCs was not influenced by 0.2 mg/ml iRoot SP. We found that the expression of osteo/odontogenic biosignatures (OPN, RUNX2, OSX, and DSPP) was significantly increased and that mineralization was significantly upregulated under SP treatment. The transcription factors for early odontogenic differentiation, namely, Runx2 and Osterix, are responsible for bone development [44, 45]. OPN plays a pivotal role at the middle stage of osteogenic differentiation [46], whereas DSPP acts as the terminal differentiation marker for odontoblasts [47]. Our finding of the upregulated master regulator for odontogenesis and osteogenesis indicates the remarkable effects of iRoot SP on the relative differentiation of BMSCs. However, in vitro research cannot reproduce the dynamic environment [48]; the effects of iRoot SP in vivo should be further studied. According to the standardization for implanted devices in contact with bone or blood, further studies of iRoot SP on genotoxicity, sensitization, and tissue implantation tests would provide a more accurate understanding of the material [49]. We believe that the randomized clinical trials of these findings should be further investigated as Clovis Mariano Faggion Jr. reported [48].

Kinases in the MAPK family are known to arouse cellular responses by transmitting extracellular signals to cells [50]. The MAPK family includes extracellular regulated kinase (ERK), c-Jun N-terminal kinase (JNK), and p38. Several stimuli, such as MagT1 [51] and dicalcium silicate microparticle- (C2S-) based biomaterials, can promote MSC differentiation through the MAPK pathway [52]. Our results indicated that their phosphorylation levels were upregulated in the presence of iRoot SP and inhibited by the specific inhibitors SB203580, SP600125, and U0126. These findings suggested that osteo/odontogenic differentiation was enhanced by iRoot SP through MAPK pathways.

The NF-*κ*B family comprises five members, namely, p65, RelB, c-Rel, p50, and p52, which play important roles in cellular proliferation, differentiation, and apoptosis [53]. In the majority of cells, NF-*κ*B proteins are localized in the cytoplasm and in connection with the inhibitor family proteins of I*κ*B. Initiation of the I*κ*B kinase (IKK) complex phosphorylates I*κ*B proteins, resulting in the polyubiquitination of I*κ*B. Subsequently, p-I*κ*B proteins are degraded by 26S proteasome, resulting in the nuclear translocation of NF-*κ*B [54]. Our results indicated that through elevation of I*κ*B phosphorylation and translocation of P65 into the nucleus, iRoot SP activates the NF-*κ*B pathway of BMSCs.

Notably, various interactions and connections exist between the NF-*κ*B pathway and the MAPK pathways. P38, JNK, and ERK are reported to phosphorylate various substrates and result in the activation of transcription factors like NF-*κ*B; in other words, NF-*κ*B is a downstream signal for MAPK [55]. It has been proposed that stimulation of the mitogen-activated protein kinase (MAP3K) family stimulates IKK activity, a key step in the NF-*κ*B cascade. For example, distinct phosphorylation of I*κ*B*α* at Ser-32 and Ser-36 is induced by the mitogen-activated protein kinase/ERK kinase kinase 1 (MEKK1) *in vivo* but the I*κ*B kinase complex *in vitro* [56]. Liu et al. reported that SB203580 inhibited the degradation of I*κ*B*α*, confirming the involvement of p38 in NF-*κ*B activation [57]. Furthermore, ERK can reduce NF-*κ*B activation via the Nrf2/HO-1 pathway [58]. NF-*κ*B induction may trigger the synthesis of JNK pathway inhibitors, implying a considerable offset relationship between NF-*κ*B and JNK [59]. However, this relationship is not completely antagonistic, as they coregulate the expression of antiapoptotic protein cIAP1 [60].

## 5. Conclusion

The present study found that 0.2 mg/ml iRoot SP-conditioned medium significantly elevated osteo/odontogenic differentiation of BMSCs via the MAPK and NF-*κ*B cascades. These findings provide a theoretical basis for clinical applications of iRoot SP.

## Figures and Tables

**Table 1:** Sense and antisense primers for real-time reverse transcription polymerase.

Genes	Primers	Sequences (5′-3′)
*RUNX2*	Forward	TCTTAGAACAAATTCTGCCCTTT
	Reverse	TGCTTTGGTCTTGAAATCACA

*OSX*	Forward	CCTCCTCAGCTCACCTTCTC
	Reverse	GTTGGGAGCCCAAATAGAAA

*ALP*	Forward	ACCTGAGTGCCAGAGTGA
	Reverse	CTTCCTCCTTGTTGGGTT

*GAPDH*	Forward	GAAGGTGAAGGTCGGAGTC
	Reverse	GAGATGGTGATGGGATTTC

## Data Availability

The datasets used and/or analyzed during the current study are available from the corresponding author on reasonable request.
